# Circuit complexity and functionality: A statistical thermodynamics perspective

**DOI:** 10.1073/pnas.2415913122

**Published:** 2025-06-02

**Authors:** Claudio Chamon, Andrei E. Ruckenstein, Eduardo R. Mucciolo, Ran Canetti

**Affiliations:** ^a^Department of Physics, Boston University, Boston, MA 02215; ^b^Department of Physics, University of Central Florida, Orlando, FL 32816; ^c^Department of Computer Science, Boston University, Boston, MA 02215

**Keywords:** thermodynamics, classical reversible circuits, quantum circuits, complexity, cryptography

## Abstract

Circuit complexity, defined as the minimum circuit size required for implementing a particular Boolean computation, is a foundational concept in computer science. Determining circuit complexity is believed to be a hard computational problem. Recently, in the context of black holes, circuit complexity has been promoted to a physical property, wherein the growth of complexity is reflected in the time evolution of the Einstein-Rosen bridge (“wormhole”) connecting the two sides of an anti-de Sitter “eternal” black hole. Here, we are motivated by an independent set of considerations and explore links between complexity and thermodynamics for functionally equivalent circuits, making the physics-inspired approach relevant to real computational problems, for which functionality is the key element of interest. In particular, our thermodynamic framework provides an alternative perspective on the obfuscation of programs of arbitrary length—an important problem in cryptography—as thermalization through recursive mixing of neighboring sections of a circuit, which can be viewed as the mixing of two containers with “gases of gates.” This recursive process equilibrates the average complexity and leads to the saturation of the circuit entropy, while preserving functionality of the overall circuit. The thermodynamic arguments hinge on ergodicity in the space of circuits which we conjecture is limited to disconnected ergodic sectors due to fragmentation. The notion of fragmentation has important implications for the problem of circuit obfuscation as it implies that there are circuits of same size and functionality that cannot be connected via a polynomial number of local moves. Furthermore, we argue that fragmentation is unavoidable unless the complexity classes NP and coNP coincide, a statement that implies the collapse of the polynomial hierarchy of computational complexity theory to its first level.

## Significance

The current paper a) defines a thermodynamic approach to the complexity of circuits of specific functionality; and b) examines the validity of the approach by introducing the notion of “ergodicity” in the space of circuits, which hinges on functionality-preserving “local mixing” models of circuit dynamics that we connect with word problems in combinatorial and geometric group theory. Our “circuit thermodynamic” framework provides an alternative paradigm for studies of circuit obfuscation, a concept in cryptography that underpins a broad range of powerful cryptographic tools.

Here we propose a physics-inspired statistical mechanics approach to the behavior of reversible computational circuits of a given functionality, in which circuit complexity—the minimal number of gates required to implement that functionality—is treated as a thermodynamic variable. While over the past decade the connection between computational complexity and thermodynamics has emerged in the course of explorations of the quantum mechanics of black-holes (1–3), our own interest in this connection was motivated by a completely different and independent line of inquiry, namely one aimed at employing statistical mechanics techniques used to diagnose irreversibility and chaos in complex physical systems in the design of cryptographic tools for protecting confidential “data-in-use”—data and any manipulations of these data—from bounded adversaries (4, 5).

By contrast to the discussion of black holes that focuses on the growth of complexity for very large random circuits and generally ignores the contribution of distinct circuits implementing the same functionality, the statistical mechanics approach presented here treats both complexity and functionality. Ultimately it is the circuit functionality—the specific computation implemented by a given circuit—that is the central object of interest in most computational problems. Our framework is based on reversible computing, which can be implemented either as permutations *P* acting on the space of $2^{n}$ strings of *n* bits, or as unitary transformations *U* acting on the $d^{n}$ dimensional Hilbert space of *n* qudits with local Hilbert space dimension *d*. For concreteness, in the body of the paper we focus on universal circuits implementing classical permutations for which the counting is discrete.^*^ Since practical implementations of quantum computation also use discrete sets of universal quantum gates to approximate continuum unitaries, the extension to quantum circuits is natural and is presented in *SI Appendix*, section A.

The first part of the paper focuses on thermodynamic arguments based on our proposed notion of “circuit ergodicity,” which implies that the space of circuits of a given size and functionality is covered uniformly and displays a “thermodynamic equilibrium” behavior analogous to that derived from a microcanonical ensemble for physical systems. Within the circuit-thermodynamics framework we establish that there are exponentially many ways to express a given functionality—a permutation *P*—in terms of reversible gates; and connect the scaling behavior as a function of complexity of two seemingly unrelated counting problems: a) how many N-gate circuits one can write for a given functionality, and b) how many distinct functionalities there are for circuits with given complexity K. The connection between these quantities is tied to the finite compressibility of typical circuits, e.g., those with gates drawn randomly from a given gate set. Finite circuit compressibility corresponds to a linear growth of complexity with number of gates (up to its maximum value exponential in *n*) with a slope less than unity, behavior that emerges as a result of functionality degeneracies.

The circuit-thermodynamics framework has a natural application to the cryptographic concept of program obfuscation—a central problem in cryptography—which, in conjunction with other cryptographic primitives, enables a tremendously powerful set of cryptographic tools. Program obfuscation is the process of “randomizing” a program while preserving its functionality. Specifically, given a computer program *P*, the process of obfuscating *P* generates another program $P^{\prime }$ that a) has a comparable size and the same functionality as *P*; and b) provides no information about *P* to an adversary with polynomial resources, except for its functionality (7). Within circuit thermodynamics, the equilibrium state defined by a microcanonical ensemble in which an exponentially large number of N-gate circuits with the same functionality appear with equal probability naturally realizes the obfuscation of every circuit in the distribution.

On a coarse-grained scale consistent with a thermodynamic treatment, the microcanonical equilibrium state is accomplished through a functionality preserving thermodynamic mixing process connecting local equilibrium states associated with “mesoscopic” subcircuits of the original circuit. This mixing process, implemented via the “flow” of gates and complexity across mesoscopic size subcircuits, is analogous to the equilibration of a set of initially separated containers of gas once the constriction between them is removed. This pathway to circuit obfuscation based on glueing together locally equilibrated subcircuits, which is conceptually different from the algebraic global-circuit approach of state of the art schemes (8–11), has recently inspired a new paradigm for cryptographic investigations of circuit obfuscation (7).

The second part of the paper returns to the issue of ergodicity in the space of circuits, a notion that we assumed in formulating our thermodynamic approach. This requires defining a dynamics in the space of circuits that enables transforming two circuits into one another while preserving size and functionality. We define a set of dynamical rules that we refer to as “*k*-string” dynamics according to which one replaces *k*-gate subcircuits by equivalent subcircuits of equal size. The resulting dynamics conserves both the functionality and size of the original circuit. We argue that, generically, such models lead to fragmentation of the space of circuits into disconnected sectors. Thus, ergodicity holds and the thermodynamic framework only applies within each sector. This conclusion raises important questions about circuit obfuscation, questions that are connected with fundamental assumptions of computational complexity theory. Finally, we propose that a natural mathematical framework for formalizing the notions of circuit collisions, fragmentation of the space of circuits, and circuit ergodicity is the word problem in geometric group theory (12, 13), a connection we plan to explore in future work.

## Results.

### Counting circuits, entropy inequalities, and the thermodynamics of circuit complexity.

As noted above, there are multiple ways of writing the same permutation *P* using reversible gates; the number of ways depends on the gate set *G* used. We define the circuit entropy

[1] $$\begin{array}{c} S\left(P,N\right)=\;\log_{2}\Omega \left(P,N\right)\;, \end{array}$$

where Ω(P,N) is the number of circuits realizing a permutation *P* with exactly N gates. The latter definition implies a) the inequality N≥K(P), where K(P) is the circuit complexity, i.e., the minimum number of gates required to implement the permutation *P*; and b) the sum-rule $\sum_{P}\;\Omega \left(P,N\right)={\left|G\right|}^{N}$, where |G| denotes the cardinality of the gate set used in the implementation of *P*.^†^ We expect that, as is the case in physical systems, our circuit thermodynamic description is universal, with details of the gate set only appearing in the values of (intensive) nonuniversal parameters.

The above counting parallels that used in the formulation of the microcanonical ensemble in statistical mechanics. In this setting, both N and the circuit functionality, i.e., the permutation *P* implemented by the circuit, are “conserved quantities.” Furthermore, we assume that all circuits implementing *P* with N gates appear with equal weight in the counting, a condition equivalent to the equal probability of microstates in the microcanonical ensemble.

A number of inequalities follow from the definition of the circuit entropy in Eq. **1**. The simplest one,

[2] $$\begin{array}{c} S\left(P_{1},N_{1}\right)+S\left(P_{2},N_{2}\right)\leq S\left(P_{1}P_{2},N_{1}+N_{2}\right)\;, \end{array}$$

expresses the fact that there may be more ways of implementing the product $P_{1}P_{2}$ than simply sequentially implementing *P*_1_ and then *P*_2_. In parallel with the entropy inequality in Eq. **2**, the circuit complexity satisfies the opposite inequality,

[3] $$\begin{array}{c} K\left(P_{1}\right)+K\left(P_{2}\right)\geq K\left(P_{1}P_{2}\right)\;, \end{array}$$

which reflects the simple fact that there may be shorter circuits implementing $P_{1}P_{2}$ than the concatenation of *P*_1_ and *P*_2_.

Using the inequality in Eq. **2**, we can immediately derive a lower bound on the entropy S(P,N) in terms of the circuit complexity K(P) of the permutation *P*:

[4] $$\begin{array}{c} S(11,N-K(P))+S(P,K(P))\leq S(P,N)\;, \end{array}$$

where 11 denotes the identity permutation, which has zero complexity, i.e., it can be expressed without using any gate. Eq. **4** can be replaced with a more useful set of bounds, namely:

[5] $$\begin{array}{cc} \left[N-K(P)\right]\;\log_{2}{\left|G\right|}^{1/2}+S\left(P,K\left(P\right)\right) & \leq S(P,N)\\ & <N\;\log_{2}\left|G\right|\;. \end{array}$$

The lower bound in Eq. **5** is composed of two contributions: The first involves the “free volume” N−K(P), with the complexity K(P) acting as an “excluded volume,” and depends on *P* only through the complexity K(P). The explicit form of this term is derived from S(11,N) via two steps: a) expressing N as $N=\sum_{\ell }a_{\ell }\;2^{\ell }$, where $a_{\ell }=0,1$ are the binary coefficients in the expansion of N in base 2 (we shall assume that N is even); and b) using [**2**] multiple times. This leads to $S\left(11,N\right)\geq \sum_{\ell }a_{\ell }\;S\left(11,2^{\ell }\right)\geq \sum_{\ell }a_{\ell }\;2^{\ell -1}S\left(11,2\right)=\frac{N}{2}S\left(11,2\right)=\frac{N}{2}\log_{2}\left|G\right|$, where we used that a two-gate identity can be written as the product of any gate *g* and its inverse $g^{-1}$. The second term on the left hand side of Eq. **4** is independent of N and accounts for the number of different possible circuits implementing the permutation *P* within the minimum possible size K(P). A simple argument, detailed in *SI Appendix*, section B for circuits composed of 3-bit gates in *S*_8_, shows that this term satisfies a lower bound proportional to K(P), which we posit to be a thermodynamic property, valid for any choice of gate set G.

Finally, the last upper bound in Eq. **5** follows immediately from the sum rule $\sum_{P}\;\Omega \left(P,N\right)={\left|G\right|}^{N}$, which implies ${\Omega \left(P,N\right)<|G|}^{N}$ and $S\left(P,N\right)<N\;\log_{2}\left|G\right|$.

Eq. **5** and the discussion accompanying it connect the microcanonical ensemble entropy to the circuit complexity and provide the foundation to what we refer to as the thermodynamics of circuit complexity.^‡^ In particular, the linear N dependence of both lower and upper bounds of S(P,N) in Eq. **5** implies that S(P,N) is extensive in N. Moreover, the assumed extensivity in K(P) of the lower bound on S(P,K(P)) motivated in the *SI Appendix*, together with the upper bound $S\left(P,K\left(P\right)\right)<(\log_{2}|G|)K\left(P\right)$ obtained from Eq. **5** evaluated for N=K(P) imply that S(P,K(P)) is extensive in K(P), a property we express as

[6] $$\begin{array}{c} S(P,K(P)\approx \gamma (P;G)\;K(P)\;, \end{array}$$

where γ(P;G) depends on the permutation *P* and the gate set, *G*, used in implementing the permutation *P*.

The main assumption of our approach, motivated by the above discussion, is that S(P,N) is an extensive function of both N and K(P)—an assumption analogous to that used in the microcanonical ensemble derivation of thermodynamics for physical systems in which the extensive thermodynamic quantities are the number of particles and the energy. An important consequence of the extensivity of the entropy with the “free volume” N−K is that the thermodynamic ensemble of N-gate circuits contains a number of circuits Ω(P,N) implementing any permutation *P* that scales exponentially with the N−K(P), behavior we will use repeatedly in what follows.

To finalize the formulation of circuit thermodynamics, we introduce the function ω(K,N) which, by contrast to Ω(P,N), which counts the number of N-gate circuits of fixed functionality, counts all N-gate circuits of fixed complexity, K:

[7] $$\begin{array}{cc} \omega (K,N) & =\sum_{P}\delta_{K,K(P)}\;\Omega \left(P,N\right)\\ & =\sum_{P}\delta_{K,K(P)}\;2^{S(P,N)}\\ & \equiv \nu \left(K\right)\;2^{\overset{¯}{S}(K,N)}\;, \end{array}$$

where $\nu \left(K\right)=\sum_{P}\delta_{K,K(P)}$ is a “density of states” that counts the number of possible functionalities (i.e., permutations) implemented by circuits of a fixed complexity, K; and $\overset{¯}{S}(K,N)$ defines an “annealed average” of S(K,N) over *P*. In turn, this allows us to write:

[8] $$\begin{array}{cc} \sigma \left(K,N\right)=\;\log_{2}\omega \left(K,N\right) & \equiv \;\log_{2}\nu \left(K\right)+\overset{¯}{S}\left(K,N\right)\;\;. \end{array}$$

The extensively of S(P,N) with N and K(P) implies that $\overset{¯}{S}(K,N)$ is itself extensive in N and K. The function $\omega \left(K,N\right)=2^{\sigma (K,N)}$, which defines the circuit complexity weight distribution for N-gate circuits, peaks at the extremum of the function σ(K,N):

[9] $$\begin{array}{c} \frac{\partial \sigma (K,N)}{\partial K}{|}_{N}=0=\frac{\partial \log_{2}\nu \left(K\right)}{\partial K}+\frac{\partial \overset{¯}{S}(K,N)}{\partial K}{|}_{N}\;. \end{array}$$

Since the entropy $\overset{¯}{S}(K,N)$ is an extensive decreasing function of complexity, ${\partial \overset{¯}{S}(K,N)/\partial K|}_{N}=-\beta \left(\kappa \right)$, where β(κ) is a positive intensive function of κ=K/N. At the extremum, $\kappa =\kappa^{*}$, $\beta \equiv \beta (\kappa^{*})$, and thus $\partial \;\log_{2}\nu \left(K\right){/\partial K|}_{K^{*}}=\beta$, implying that $\nu \left(K^{*}\right)=2^{\beta K^{*}}$ increases exponentially with complexity, consistent with the counting estimates for the number of circuits with given complexity K (1). At the extremum, Eq. **8** assumes the form

[10] $$\begin{array}{c} \sigma \left(K^{*},N\right)=\beta \;K^{*}+\overset{¯}{S}\left(K^{*},N\right)\;\;. \end{array}$$

It is gratifying that Eq. **10** can be recast in a form familiar from the thermodynamics of physical systems: If we interpret K as the negative of the energy, E=−K (or equivalently, ϵ=E/N=−κ) and β=1/T as the inverse of the temperature *T*, Eq. **10** can be rewritten in terms of the equilibrium free energy F(T,N),

[11] $$\begin{array}{c} -T\;\sigma_{e}\left(E,N\right)\equiv F\left(T,N\right)=E-T\;{\overset{¯}{S}}_{e}\left(E,N\right)\;, \end{array}$$

where subscripts *e* represent the respective *σ* and $\overset{¯}{S}$ functions evaluated at the corresponding negative energies. Within this correspondence, the smallest (large negative) energy state—corresponding to high complexity—would represent a low entropy solid, while the largest (zero) energy state—corresponding to low complexity—would represent a high entropy gas.

The direct analogy with statistical mechanics can be exploited further in the calculation of the probability distribution of complexities for N-gate circuits:

[12] $$\begin{array}{c} P_{N}\left(K\right)=\frac{\omega (K,N)}{\sum_{K=0}^{N}\omega \left(K,N\right)}\;, \end{array}$$

where the form of ω(K,N) can be obtained by expanding $\sigma \left(K,N\right)=\;\log_{2}\omega \left(K,N\right)=\beta K+\overset{¯}{S}\left(K,N\right)$ to second order in $\Delta K=(K-K^{*})$, the departure from the solution of the extremum condition:

[13] $$\begin{array}{cc} \log_{2}\omega \left(K,N\right) & =\overset{¯}{S}\left(K^{*},N\right)+\beta K^{*}\\ & \;+\frac{\text{1}}{2}\;{\Delta K}^{2}\;\frac{\partial^{2}\overset{¯}{S}\left(K,N\right)}{\partial K^{2}}{|}_{N,K=K^{*}}+\cdots \;\;. \end{array}$$

Using the extensivity of the entropy in K and N we can write $\overset{¯}{S}\left(K^{*},N\right)=-\beta K^{*}-\beta \mu N+\cdots$, where we have borrowed the statistical mechanics notation involving the equilibrium “chemical potential” *μ*. At the extremum, this leads to the simplification of the first two terms in Eq. **13** to $\overset{¯}{S}\left(K^{*},N\right)+\beta K^{*}=-\beta \mu N$ from which it then follows that:

[14] $$\begin{array}{cc} \omega (K,N) & =2^{-\frac{1}{2NT^{2}c_{N}}{\Delta K}^{2}}\;2^{-\beta \mu N}\;. \end{array}$$

Thus, the probability distribution in Eq. **12** is a Gaussian peaked at $K^{*}=\kappa^{*}N$ linear in N with a width (rmsd) $\Delta_{\mathrm{rms}}\propto \sqrt{NT^{2}c_{N}}$, where $c_{N}=-\partial K/\partial T{|}_{K^{*},N}$ is a positive intensive quantity analogous to the specific heat in thermodynamics, which, in physical systems measures the increase in energy induced by an increase in temperature and controls energy fluctuations around the thermal equilibrium state at temperature *T*. Finally, Eq. **14** and the sum rule $\sum_{P}\;\Omega \left(P,N\right)=\sum_{K=0}^{N}\omega \left(K,N\right)={\left|G\right|}^{N}$ determines the leading behavior of the chemical potential, $\mu =-T\log_{2}\left|G\right|$.

It is important to stress that we expect that generic solutions of the extremum condition for $\kappa^{*}$, which depend on the gate set through the value of *β*, are neither 0 nor 1 but lie in between, $0<\kappa^{*}<1$. This expectation underscore two conclusions that we reach by generic thermodynamic arguments alone, namely that: (a) the average complexity grows linearly with the depth of the circuit, recovering results obtained by explicit calculation (14, 15); and more specifically that (b) typical circuits display a finite circuit compressibility with a compression factor $\eta =(1-K^{*}/N)$, with 0<η<1. We stress that these results rely on an important and nontrivial feature that emerges from the thermodynamic arguments at the root of Eq. **9**, namely the balance between two competing effects: the exponential increase in the density of states $\nu \left(K\right)\approx 2^{\beta K}$ and the decrease in the entropy $\overset{¯}{S}(K,N)$ with increasing K. We also note that, while our extensivity assumption for $\log_{2}\nu \left(K\right)$ and the linear increase of the average complexity with circuit depth should hold up to a maximum complexity exponential in *n*, $K_{\max}∼n\;2^{n}$, our focus is on polynomial (in *n*) size circuits with N>>n.^§^ In *SI Appendix*, section C, we present a model for the growth of complexity of a random circuit, which accounts for functionality degeneracy, and recovers results obtained from the thermodynamic treatment that relies on the extensivity of $\overset{¯}{S}(K,N)$ and σ(K,N) with N and K.

**Fig. 1.**
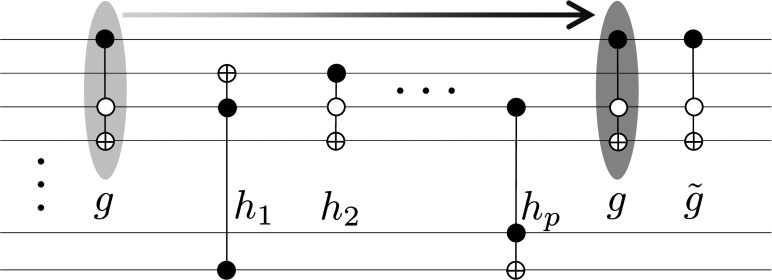
An example of “pushing” a Toffoli gate *g* past gates $h_{1},h_{2},\cdots ,h_{p}$ so as to annihilate *g* with its inverse $\tilde{g}$ (identical to *g* for a Toffoli gate), thereby reducing the circuit size by two gates. Typically, in a random circuit over *n* bitlines, a Toffoli gate can travel past O(n) gates with which it commutes before encountering either a gate with which it does not commute or, with probability $O(1/n^{3})$, its inverse. The process depicted leads to a compression of the circuit size by a factor $\left(1\;\;\text{to}\;2\xi /n^{2}\right)$, where *ξ* is a constant of O(1).

To further motivate the notion that generic circuits display a finite compressibility with a compression factor 0<η<1, we consider the following scenario leading to a lower bound on *η*. Consider a random circuit of 3-bit Toffoli gates and imagine “pushing” a gate through the circuit until the gate encounters either i) a gate with which it does not commute, in which case we stop; or ii) its inverse, in which case the pair (the gate and its inverse) annihilate, decreasing the size of the circuit by two gates (Fig. 1).^¶^ For a Toffoli gate, the probability that it does commute with a gate on its path is of the order 1−O(1/n), or equivalently, a gate can be “pushed” through O(n) gates before either stopping as in case i) or annihilating as in case ii). The overall probability of annihilation is $O(1/n^{2})$, accounting for the probability $O(1/n^{3})$ that the inverse is met in any of the O(n) attempts before stopping. Hence, this process leads to a compression of the circuit by a factor $\left(1\;\;\text{to}\;2\xi /n^{2}\right)$ of its original size, where *ξ* is a constant of O(1). This implies that circuits with greater than $O(n^{2})$ universal (Toffoli) gates are compressible with $\eta =2\xi /n^{2}$, setting a lower bound for compressibility of random circuits of universal gates. Indeed, since the probability of annihilation of linear gates—NOTs and CNOTs—scales more favorably as 1/n and $1/n^{2}$ respectively, circuits composed of gates from the universal set of Toffolis, CNOTs, and NOTs are more compressible with a compression factor *η* above the Toffoli bound. [It is worth mentioning that since any linear circuit can be implemented with $O(n^{2})$ gates, purely linear circuits are highly compressible, with $\eta ∼(1-n^{2}/N)$.]

Finally, we note that, as already alluded to in the introduction, a compression factor 0<η<1 also implies that functionality degeneracies are nonnegligible, a conclusion which one could have reached already from Eqs. **4** and **5**, which highlight the fact that there are exponentially many circuits that realize any permutation *P*.

### Thermodynamic mixing of circuits—an application to circuit obfuscation.

As we have learned from physical systems, thermodynamics provides a platform for harnessing heat into useful work or for analyzing thermodynamic processes that transfer heat/entropy or molecular species. We find it reassuring that, in the context of circuits, this simplest of thermodynamic concepts—the entropy of mixing—provides a potentially important application to circuit obfuscation. As already alluded to in the introduction, obfuscation of circuits follows immediately from two tenets of circuit thermodynamics: i) the fact that there are exponentially many comparable size circuits which implement the same functionality; and ii) that in the fully mixed maximum entropy state for an ensemble of such circuits is described by a uniform (microcanonical) distribution, i.e., drawing a circuit from this distribution results in any one of the exponentially many Ω(P,N) realizations of the N-gate circuit with functionality *P* with equal probability. Thus, any two different circuits of the same size and functionality drawn from the microcanonical ensemble of circuits cannot be distinguished from one another by an adversary with polynomial resources—the defining condition for the concept of circuit obfuscation.

Given an N-gate circuit of a given functionality, one can ask: How can one iteratively randomize the gate makeup of a circuit while preserving its size and functionality in order to realize circuit obfuscation in the sense defined above? Strictly speaking, the microcanonical assumption, namely that all N-gate circuits of a given functionality *P* appear with equal weight in the count Ω(P,N), hinges on “ergodicity” in the space of circuits of a given functionality, a concept which implies a “microscopic” dynamical process and “equilibration” of collections of gates in a circuit, analogous to the thermalization induced by microscopic collisions of atoms or molecules in a gas. We shall return to the complex and interesting question of microscopic dynamics below. Here, we concentrate on a coarse-grained model for thermodynamic mixing on a “macroscopic” scale. We will imagine that by some process, which we will outline in the next section of the paper, we can divide the circuit into *M* smaller “mesoscopic” size subcircuits, and that we are able to fully equilibrate each of these subcircuits. Each of these “mesoscopic” subcircuits are assumed to be large enough to obey the laws of circuit thermodynamics introduced above but small enough so that an appropriate set of “microscopic” dynamical rules leads to equilibration in a time *τ*_*eq*_.

**Fig. 2.**
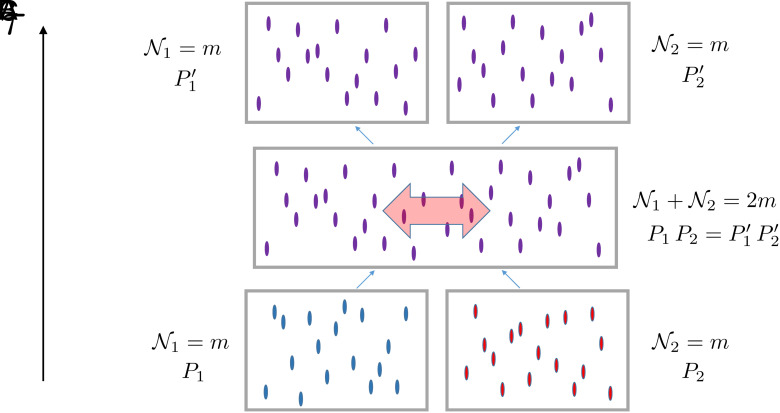
The elementary C-gate represents the equilibration between two circuits of equal sizes $N_{1}=N_{2}=m$. The two individual *m*-gate circuits, depicted in (*A*), have functionalities *P*_1_ and *P*_2_, respectively. In (*B*) they are brought into contact and exchange gates and functionality, realizing a combined functionality $P_{1}P_{2}$, while preserving the total number of gates $N_{1}+N_{2}=2m$. In (*C*), following equilibration [symbolized by the red double-arrow in (*B*)] the 2m-gate circuit is split in the middle such that each of the partitions contains *m* gates and represents, respectively, functionalities $P_{1}^{\prime }$ and $P_{2}^{\prime }$, with $P_{1}^{\prime }\;P_{2}^{\prime }=P_{1}\;P_{2}$. The thermodynamic process defining the C-gate increases the entropy.

The equilibration of an arbitrary (polynomial size) circuit is then established by connecting a string of short *m*-gate segments/subcircuits, More precisely, consider the situation depicted in Fig. 2, in which two *m*-gate subcircuits of functionality *P*_1_ and *P*_2_, respectively, are allowed to exchange gates and functionality via some dynamical rules. Thermalization at the mesoscopic scale implies that, after a time scale *τ*_*eq*_, a) the counting of individual subcircuits (Fig. 2*A*) satisfy the microcanonical assumption, and b) the concatenated circuit with 2m gates and functionality $P_{1}P_{2}$ satisfies the thermodynamic inequalities Eqs. **2** and **3**.

**Fig. 3.**
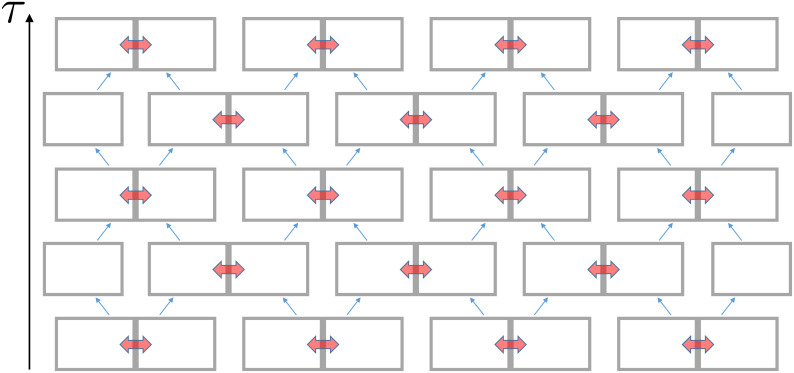
A brickwall C-circuit that progressively expands the “mesoscopic” (local) equilibrium established between pairs of *m*-gate subcircuits (depicted by the gray boxes) into a thermodynamically mixed equilibrium state for the full circuit, while preserving the functionality of the original concatenation of gates. Neighboring *m*-gate subcircuits are brought into local thermal equilibrium via the exchange (depicted by the two-headed red arrows) of gates and functionality (while preserving both the number of gates and the combined functionality of the subcircuits). A subcircuit is paired with either its neighbor to the *Left* or to the *Right*, alternating in each time step (following the pattern of blue arrows).

Given the mesoscopic thermalization assumption, which we will revisit below, we are now in position to define a coarse-grained model for thermodynamic mixing that takes as input a circuit *C* that is split into *M* subcircuits, each composed of m=N/M gates: $C=c_{1}c_{2}...c_{M}$. A subcircuit *c*_*i*_ (i=1,⋯,M) can be thought of as a degree of freedom in a *d*-dimensional space with $d={\left|G\right|}^{m}$ states, i.e., a dit; and thus a circuit can be viewed as a string of *M* such dits. The coarse-grained mixing of the full circuit *C* is implemented as a circuit acting on dit-strings, i.e., a “circuit acting on circuits”—hereafter referred to as a C-circuit—built out of gates acting on dits, i.e., “gates acting on circuits”—referred to as C-gates. Fig. 3 depicts a brickwall C-circuit of C-gates acting on a pair of neighboring dits $c_{i-1}\left(\tau \right)$ and $c_{i}\left(\tau \right)$ in layer *τ* and evolving them into $c_{i-1}\left(\tau +1\right)$ and $c_{i}\left(\tau +1\right)$ after one “time” step, i.e., into layer *τ* + 1 of the C-circuit. The brickwall C-circuit is a scrambler of circuits, much as usual brickwall circuits of dit- or qudit-gates are scramblers of states of dits or qudits (see for example ref. 16 and references therein).

The action of an individual C-gate, which takes place on a time scale longer than *τ*_*eq*_, is based on the mesoscopic equilibration assumption, and is implemented in three steps that parallel those depicted in Fig. 2, as follows: a) take $c_{i-1}\left(\tau \right)$ and $c_{i}\left(\tau \right)$, with functionalities $P(c_{i-1}\left(\tau \right))$ and $P(c_{i}\left(\tau \right))$; b) draw a circuit $c_{\mathrm{aux}}$ uniformly out of the Ω(P,2m)2m-gate circuits with functionality $P=P\left(c_{i-1}\left(\tau \right)\right)\;P\left(c_{i}\left(\tau \right)\right)$; and c) split the 2m-gate circuit $c_{\mathrm{aux}}$ into two *m*-gate circuits $c_{i-1}\left(\tau +1\right)$ and $c_{i}\left(\tau +1\right)$. We note that the action of a C-gate on the two dits $c_{i-1}$ and *c*_*i*_ preserves functionality of the product of the two associated subcircuits, a “conservation law” that maintains the functionality of the overall circuit. (We also note that the stochastic process defined above could be replaced by the action of a C-circuit built from deterministic C-gates with given substitution truth tables chosen randomly.)

As alluded to above, the action of the brickwall C-circuit progressively expands the “local” equilibrium within each of the *m*-gate subcircuits into a thermodynamically mixed equilibrium state for a full circuit *C* of any size N. As already alluded to above, the equilibration process induced via the C-circuit is analogous to the equilibration of connected thermodynamic systems (e.g., containers of gas molecules) that were initially isolated from one another. More specifically, this thermodynamic mixing of circuits reflects three properties of the explicit C-circuit implementation: i) the circuit entropy after the application of each C-gate never decreases, but increases or remains the same; ii) through subsequent layers of the scrambling process, functionalities of individual subcircuits change but the functionality of the overall concatenated circuit is preserved; and, most importantly, iii) the thermalization in the space of dits defining the action of a stochastic C-gate and the layer-by-layer evolution of the circuit leads to the branching into a multitude of paths, which implies that memory of the initial circuit is lost, i.e., the scrambling process is irreversible.

Given the one-dimensional brickwall arrangement of gates acting on *M* dits, such as the C-circuit in Fig. 3, the number of layers required for scrambling the initial dit-string (in our case, the initial circuit *C*) should scale as $M^{\gamma }$. In random circuits acting on dits without conservation laws *γ* = 1 (16–19) while in the presence of locally conserved quantities we expect *γ* ≥ 2 (20–22). For the case of scrambling by C-circuits, a C-gate acting on two subcircuits of functionalities *P*_1_ and *P*_2_, respectively, may change *P*_1_ and *P*_2_ but preserves $P_{1}P_{2}$. This more complicated “multiplicative” (rather than additive) conservation law has not yet been analyzed in detail, but we expect that both the saturation of the entropy to its maximum attainable value and the state of uniform average complexity, $\overset{¯}{K_{i}}=K(P)/M$, for each of the *M* subcircuits of a C-circuit *C* are reached within a time polynomial in *M*. The above arguments imply that once any two initially distinguishable circuits of the same size and functionality are processed through a polynomial number of layers of a C-circuit, they become indistinguishable from one another by any adversary with polynomial resources.

We note that the general line of reasoning presented so far makes certain assumptions, some of which will be challenged below. In particular, the notion of fragmentation of the space of circuits of a given size and functionality into disconnected sectors, which we introduce shortly, will restrict the thermodynamic framework to individual sectors. Clearly, fragmentation of the space of circuits will require sharpening of our definition of circuit obfuscation.

### Microscopic dynamics of circuits.

All thermodynamics-based arguments presented thus far rely on the assumption of ergodicity, namely that some microscopic dynamical rules that connect circuits of same size and functionality lead to a uniform covering of the space of all such circuits. Moreover, we assumed that equilibration across the space of circuits is achieved in polynomial time, i.e., that, given the dynamical rules, connecting any two circuits can be achieved with a number of steps that scales polynomially in the number of gates, N. This assumption raises a number of interesting and up to now unexplored questions.

**Fig. 4.**
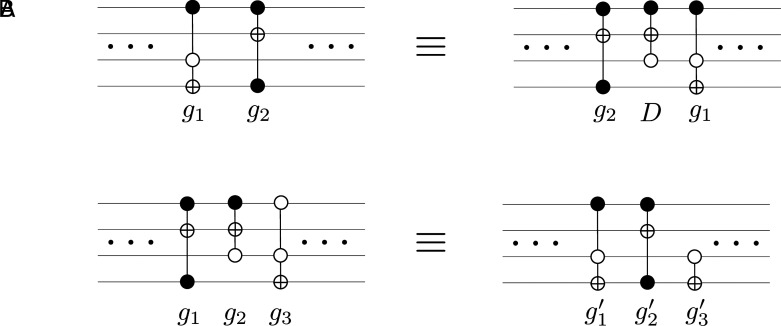
(*A*) An example of a collision (the interchange) $g_{1}\;g_{2}↔g_{2}\;D\;g_{1}$, where the debris gate *D* is needed to preserve the functionality of the initial two-gate segment of the circuit. (*B*) An example of a substitution of a segment with *k* = 3 gates, $g_{1}\;g_{2}\;g_{3}↔g_{1}^{\prime }\;g_{2}^{\prime }\;g_{3}^{\prime }$. The same arrangements in (*B*) can be used as examples of two functionally equivalent circuits with N=3 that cannot be connected via a *k* = 2 substitution rule.

To begin with, by contrast to motion of molecules in a gas in the course of collisions, which is governed by physical laws, there is no unique or natural dynamics for moving/colliding gates in a circuit in ways that preserve functionality. A naive notion of “gate collisions,” analogous to collisions of gas particles, must take into account the noncommutative algebra of gates in a universal set. If one defines a gate collision as an interchange of gates *g*_1_ and *g*_2_ acting on shared bitlines, then preserving functionality before and after the collision implies, generically, a substitution $g_{1}\;g_{2}↔g_{2}\;D\;g_{1}$, where *D* is a “debris” gate needed so that algebraically $g_{1}\;g_{2}=g_{2}\;D\;g_{1}$. An example of such a collision is illustrated in Fig. 4*A* for two Toffoli gates. A macroscopic number of such debris-generating collisions would inevitably lead to an irreversible increase in the size of the circuit, violating the constraint of a fixed number of gates.

A more fruitful direction is to define a dynamics in the space of circuits based on gate-substitution rules that exchange a string of gates with an alternate string with same size and functionality. One can view an N-gate circuit as a quasi-1D system, or a chain of N sites, in which a gate *g*_*i*_ (a non-Abelian group element) is placed at each site *i*. Global functionality is determined by $P=g_{1}\;g_{2}\cdots g_{N}$, and a local microscopic dynamics must preserve this overall functionality. The functionality-preserving local dynamical model we have in mind involves the following substitution of a string of *k* consecutive gates:

[15a] $$\left(g_{i},g_{i+1},\cdots ,g_{i+k-1}\right)↔\left({g^{\prime }}_{i},{g^{\prime }}_{i+1},\cdots ,{g^{\prime }}_{i+k-1}\right)$$

[15b] $$g_{i}g_{i+1}\cdots g_{i+k-1}\;\;=\;g_{i}^{\prime }g_{i+1}^{\prime }\cdots g_{i+k-1}^{\prime }\;.$$

An example of a *k* = 3 circuit identity involving Toffoli and CNOT gates is shown in Fig. 4*B*. Substitution rules for fixed (and small) *k* can be built from a catalog of strings of *k* gates that multiply to the same permutation. Transition probabilities among the *k*-length strings, in the case the catalog is exhaustive, can be chosen to be

[16] $$\begin{array}{cc} & T_{\left(g_{i},\cdots ,g_{i+k-1}\right),\left(g_{i}^{\prime },\cdots ,g_{i+k-1}^{\prime }\right)}\\ & =\frac{1}{\Omega (g_{i}\;\cdots \;g_{i+k-1},k)}\;\delta_{g_{i}\;\cdots \;g_{i+k-1},g_{i}^{\prime }\;\cdots \;g_{i+k-1}^{\prime }}\;. \end{array}$$

We note that the stochastic C-gate used above can be implemented via such a transition matrix element with k=2m. Alternatively, one can dilute the connectivity associated with the *T*-matrix so that not all pairs of “*k*-strings” satisfying Eq. 15 are connected via a matrix element. We note that since the number of circuits with *k* gates is ${\left|G\right|}^{k}$, enumerating the equivalence rules for large *k* becomes prohibitive.

While to our knowledge this type of dynamical model has not been discussed in the literature and a detailed study of the model is outside the scope of this paper, we can already point to a set of fundamental issues that have important implications for the discussion of circuit thermodynamics. In particular, our intuition suggests that the space of circuits with functionality $P=g_{1}\;g_{2}\cdots g_{N}$ evolving via *k*-range rules will generically fragment into a number of disconnected sectors. A simple example that supports the notion of fragmentation is to consider a functionality-preserving dynamics that only connects 2-strings if and only if two neighboring gates *g*_*i*_ and $g_{i+1}$ commute, in which case we exchange $\left(g_{i},g_{i+1}\right)↔\left(g_{i+1},g_{i}\right)$ with probability 1/2. This dynamics allows a gate to move left and right through the list of gates in the circuit by passing other gates with which it commutes, but not past those gates with which it does not commute. This dynamics preserves the number of gates of each type in the circuit and thus does not allow one to connect the two equivalent circuits in Fig. 4*B*. A less restricted dynamics with *k* = 2 in this same example would still not allow the two sequences of three gates in Fig. 4*B* to be connected.

Fragmentation implies that a particular dynamics is ergodic only within individual sectors, and thus all the thermodynamic results would apply, but only within each disconnected sector. Implicit in this statement is that, within a given sector, ergodicity is reached within a number of steps defining the particular dynamics that is polynomial in N. In this case, the finite compressibility of generic circuits is an example of a property that survives in the fragmented system, where the compression factor should be determined by some weighted average over fragments.

The intuition about fragmentation and polynomial thermalization is supported by computational complexity theory. Connecting two circuits of the same size and functionality by a polynomial number of local, functionality-preserving moves would imply NP = coNP, contradicting widely accepted beliefs of computational complexity theory. To understand this statement, recall that the complexity class NP contains YES decision problems that can be verified in polynomial time, while the class coNP contains problems for which NO solutions can be verified in polynomial time. If two circuits can be connected via a polynomial sequence of moves, Circuit Equivalence, a problem in coNP, is then also in NP because the sequence is itself a witness for the YES decision. Similarly, connecting two circuits through a polynomial sequence of moves implies that Circuit Inequivalence, a problem in NP, is also in coNP. Moreover, it is also well known that Circuit Equivalence and Circuit Inequivalence are among the hardest problems in their respective coNP and NP classes, i.e., they are in classes coNP-complete and NP-complete, respectively. “Completeness” indicates that any problem in coNP or NP can be reduced, respectively, to Circuit Equivalence or Circuit Inequivalence in polynomial time. Given that a polynomial number of moves placed Circuit Equivalence in NP and Circuit Inequivalence in coNP the conclusions of the above line of argumentation is that NP = coNP, implying that fragmentation of the space of N gate circuits implementing a permutation *P* is inevitable, regardless of whether the sequence of moves is easy or hard to find.

Clearly, fragmentation and the accompanying broken ergodicity significantly alters the IO construction presented above. For a system with multiple sectors, the relevant question becomes: Given two circuits *C*_1_ and *C*_2_, can one decide in polynomial time whether they belong to the same ergodic (thermalized) sector or not? Physical intuition based on the scrambling of information, irreversibility, and chaos in closed systems with a large number of degrees of freedom leads to a natural conjecture that, for nontrivial dynamical rules, this is a hard (NP) decision problem. If this is the case, then the thermodynamic framework does in fact provide a path to Indistinguishability Obfuscation of any two circuits, *C*_1_ and *C*_2_. Otherwise the thermodynamic framework could only establish IO for circuits in the same sector.

## Discussion and Future Directions.

This paper presents a thermodynamic framework for describing course-grained properties of large N-gate reversible classical (and quantum) circuits with N≫n (with N polynomial in *n*, the number of bitlines of the circuit) and a given functionality, defined by the permutation *P* implemented by the circuit. Our construction of circuit thermodynamics is based on three assumptions that underpin the logical consistency of the approach: i) the functionality *P* only appears through the circuit complexity K(P), i.e., the minimum number of gates required for the implementation of the permutation *P*; ii) the entropy defined by counting of the number of possible N-gate circuits implementing *P* is extensive in N and K(P); and iii) ergodicity in the space of circuits, which as a result of fragmentation can only occur in disconnected sectors, requires a “time” (i.e., number of dynamical moves) that is polynomial in N, the size of the circuit.

The fragmentation of the space of circuits suggests a number of questions we expect to address through more detailed analytical and computational studies of the “*k*-string” dynamics of reversible circuits: i) is there is a critical value *k*_*c*_ such that if $k_{c}\leq k\leq N$ the space of circuits of size N and functionality *P* becomes fully connected, and how does this value scales with the number of bitlines *n*? ii) If the space is fragmented, how does the number of fragments scale with *k* and N and (possibly) the complexity of *P*? iii) can one make more precise statements about the hardness of deciding whether any two circuits belong to the same or different sectors? We note that even though we raise these questions in the context of the permutation group $S_{2^{n}}$, the thermodynamic framework and the issues it raises can be generalized to other groups.

In summary, the thermodynamic perspective to complexity and functionality of circuits proposed here charts an unexplored line of inquiry. Developing this direction further and addressing quantitatively the questions it raises will require combining the intuition and tools of theoretical physics with mathematical ideas and techniques from the theory of classical and quantum computation. We believe that this work provides a framework that should stimulate fresh ways of thinking and fruitful interactions at the interface between physics and computer science. In particular, the issue of fragmentation which, to our knowledge, has not been explored by computer scientists, is a topic of much current interest to the physics communities working on classical and quantum dynamics (23–27). Conversely, the problem of dynamics of systems with “multiplicative” rather than additive conservation laws (as is the case with the functionality of circuits) defines a new intriguing problem for theoretical physicists interested in classical and quantum dynamics.

We close by suggesting a direction that formalizes many of the ideas presented above, especially those related to the dynamics and ergodicity of systems of reversible circuits. The proposed direction starts from the observation that gate-based reversible classical and quantum computations are naturally framed in the language of group theory: Gates represent group generators and the “*k*-string” substitution rules can be regarded as relations that define a group presentation. Thus, the “*k*-string” dynamics defined in this work is intimately connected to the word problem (12, 13) of combinatorial and geometric group theory. This connection points to a formal mathematical framework that we expect will provide tools to further develop the circuit thermodynamics approach proposed here.

## Supplementary Material

Appendix 01 (PDF)

## Data Availability

All study data are included in the article and/or *SI Appendix*.

## Footnotes

^*^We note that here we focus on even permutations in the alternating group $A_{2^{n}}$, which can be decomposed into products of universal 3-bit gates (6). The realization of odd permutations requires either one additional *n*-bit gate or one ancilla bit. More generally, the group $S_{2^{n}}$ can be embedded in the alternating group $A_{2^{n+1}}$, an embedding that formalizes the incorporation of ancillas, and which also suggests that, given a computational model, the thermodynamic discussion presented here for even permutation extends to odd permutations without essential changes.

^†^For 3-bit gates in *S*_8_, $\left|G\right|=8!\left(\begin{array}{c} n\\ 3 \end{array}\right)$ and for gates in the universal set (NOT, CNOT, Toffoli), |G|=n+n(n−1)+n(n−1)(n−2)/2.

^‡^In analogy to statistical physics, by introducing weights for different gates one could have also constructed a canonical ensemble for circuits.

^§^The circuit entropy $\overset{¯}{S}$ continues to grow linearly with N even when N increases beyond the maximum circuit complexity. We note that, if growth of the Einstein-Rosen wormholes is unbounded, circuit entropy could describe that growth.

^¶^Note that here we are considering random circuits built gate-by-gate, as opposed to minimum circuits describing random permutations. The former are compressible while the latter are not.
