# Supplementary material for: Circuit complexity and functionality: A statistical thermodynamics perspective
Source: Proc Natl Acad Sci U S A. 2025 Jun 2;122(23):e2415913122. doi: 10.1073/pnas.2415913122 (PMC12168019; doi:10.1073/pnas.2415913122)
Supplement: Supplementary file 1 — Appendix 01 (PDF) [file pnas.2415913122.sapp.pdf]

Supporting Information for

Circuit complexity and functionality: a statistical thermodynamics perspective

Claudio Chamon, Andrei E. Ruckenstein, Eduardo R. Mucciolo, Ran Canetti

This PDF file includes:

Supporting text

Supporting Information Text

A - Extension to quantum circuits. The extension of the statistical thermodynamics approach to quantum circuits can proceed via two routes that employ: (1) gates defined over a continuous unitary group such as, for example, 2-qubit gates drawn from a Haar-uniform measure on $U(4)$; or (2) a discrete set of universal gates* such as CNOTs, Hadamard gates H, and T gates ($\pi/8$ phase gates), or some other discrete choice of an entangling 2-qubit gate and gates that approximate arbitrary 1-qubit rotations (?). For the purposes of this discussion we choose option 2, which is both simpler and more relevant to standard implementations of quantum computation.

We start by extending the expression for the number of circuits realizing a permutation P with $\mathcal{N}$ gates drawn from a universal set of classical reversible gates, G,

$$\Omega(P,\mathcal{N}) = \sum_{\{g_1,g_2,\dots,g_\mathcal{N}\}\in G^\mathcal{N}} \delta_{P,g_1\,g_2\,\dots\,g_\mathcal{N}}\ , \qquad [1]$$

to the quantum case for which gates $u_1, u_2, \dots, u_\mathcal{N}$ are drawn from a discrete universal set of quantum gates, G_Q,

$$\Omega_\epsilon(U,\mathcal{N}) = \sum_{\{u_1,u_2,\dots,u_\mathcal{N}\}\in G_Q^\mathcal{N}} \delta_\epsilon(U, u_1\, u_2\, \dots\, u_\mathcal{N})\ , \qquad [2]$$

where

$$\delta_\epsilon(U,V) = \begin{cases} 1, & \text{if } d(U,V)\le\epsilon \\ 0, & \text{if } d(U,V)>\epsilon \end{cases}\ , \qquad [3]$$

and $d(U,V)$ is a metric distance on the space of unitaries. The distance between the two unitaries measures how well V approximates U, i.e., $d(U,V)$ quantifies the approximation error. Approximating a unitary as a product of gates in a discrete set is commonplace in quantum computation (?). Examples of metrics quantifying the approximation error are $d(U,V) = \max_{|\psi\rangle} ||(U-V)\,|\psi\rangle||$ or $d_{\rm tr}(U,V) = \arccos\left(\frac{1}{D_H}|{\rm tr}\, U^\dagger\, V|\right)$, where D_H is the dimension of the Hilbert space on which the unitaries act. (For example, the former metric is used in Ref. (?) and the latter in Ref. (?).) The metric satisfies the inequality

$$d(U_1U_2, V_1V_2) \le d(U_1,V_1) + d(U_2,V_2)\ , \qquad [4]$$

which leads us to infer that $\delta_{\epsilon_1}(U_1,V_1)\ \delta_{\epsilon_2}(U_2,V_2) = 1$ implies $\delta_{\epsilon_1+\epsilon_2}(U_1U_2,V_1V_2)=1$ (the reverse is not necessarily true). Using these relations we can write

$$\Omega_{\epsilon_1}(U_1,\mathcal{N}_1)\ \Omega_{\epsilon_2}(U_2,\mathcal{N}_2) \le \Omega_{\epsilon_1+\epsilon_2}(U_1U_2,\mathcal{N}_1+\mathcal{N}_2)\ . \qquad [5]$$

We proceed by defining a quantity

$$\bar\Omega_\delta(U,\mathcal{N}) \equiv \Omega_{\delta\mathcal{N}}(U,\mathcal{N}), \qquad [6]$$

for which the choice of ϵ scales with $\mathcal{N}$ via an infinitesimal constant δ, chosen so that the error $\epsilon = \delta\,\mathcal{N}$ is small for circuits of a bounded size (e.g., polynomial on the number n of qubits). †

Eq. (5) then translates into an inequality for $\bar\Omega_\delta$,

$$\bar\Omega_\delta(U_1,\mathcal{N}_1)\ \bar\Omega_\delta(U_2,\mathcal{N}_2) \le \bar\Omega_\delta(U_1U_2,\mathcal{N}_1+\mathcal{N}_2)\ , \qquad [7]$$

from which we derive the inequality for the entropy $\mathcal{S}_\delta(U,\mathcal{N}) = \log_2\bar\Omega_\delta(U,\mathcal{N})$, analogous to that satisfied by the entropy permutation gates, namely,

$$\mathcal{S}_\delta(U_1,\mathcal{N}_1) + \mathcal{S}_\delta(U_2,\mathcal{N}_2) \le \mathcal{S}_\delta(U_1U_2,\mathcal{N}_1+\mathcal{N}_2)\ . \qquad [8]$$

We can similarly extend the defininion of complexity to account for the approximation error:

$$\mathcal{K}_\delta(U) \equiv \min_\mathcal{N}\left\{\mathcal{N}\ \middle|\ \bar\Omega_\delta(U,\mathcal{N})>0\right\}\ . \qquad [9]$$

It then follows from Eq. (9) and the inequality Eq. (7) that

$$\mathcal{K}_\delta(U_1) + \mathcal{K}_\delta(U_2) \ge \mathcal{K}_\delta(U_1U_2)\ . \qquad [10]$$

*The discrete gate sets are chosen so that the inverses of all gates in the set are also included.

†We note that the issues discussed above concerning the need of introducing the "tolerance", ϵ, in the definition of Ω_ϵ in Eq. (2) has a direct analogue in physical systems. Specifically, when defining a microcanonical potential for a physical system, $\Omega^{\rm ph}_\epsilon(E,N)$, which counts the number of microstates of given energy E and particle number N, it is necessary to bracket states of energy between $E-\epsilon/2$ and $E+\epsilon/2$, defining an interval of size ϵ around the value of E. The inequality $\Omega^{\rm ph}_{\epsilon_1}(E_1,N_1)\ \Omega^{\rm ph}_{\epsilon_2}(E_2,N_2) \le \Omega^{\rm ph}_{\epsilon_1+\epsilon_2}(E_1+E_2,N_1+N_2)$ then follows. Two comments are in order: first, notice that choosing $\epsilon=\delta\,N$ is natural as it keeps the intensive energy E/N within a fixed interval of size δ. Second, the actual choice of the ϵ is immaterial in statistical mechanics in the thermodynamic limit, because ϵ corresponds to the thickness of an energy shell in a N-dimensional space, and therefore the correction to the entropy from that shell is subextensive. As a result, details on the specific choice of ϵ (beyond the definitional need to introduce it) are inconsequential.

With these definitions for $S_\delta(U,\mathcal{N})$ and $\mathcal{K}_\delta(U)$, which incorporate the approximation errors in realizing unitaries in terms of a discrete set of universal quantum gates, the thermodynamic approach for quantum circuits mirrors that presented in the body paper for reversible classical circuits.

Microscopic dynamics of quantum circuits: The local dynamical rules introduced in the context of classical reversible circuits, can be easily extended to quantum circuits. In the case of classical permutation gates, the functionality-preserving local dynamical model involves substitution of strings of k consecutive permutation gates (denoted by g),

$$(g_i, g_{i+1}, \dots, g_{i+k-1}) \longleftrightarrow \left(g_i', g_{i+1}', \dots, g_{i+k-1}'\right) \qquad [11a]$$

$$g_i\, g_{i+1}\, \dots\, g_{i+k-1} \;=\; g_i'\, g_{i+1}'\, \dots\, g_{i+k-1}' \,. \qquad [11b]$$

In the quantum case the rules can be generalized in a natural way to account for the approximation error in the substitution of strings of k unitary gates (denoted by u):

$$(u_i, u_{i+1}, \dots, u_{i+k-1}) \longleftrightarrow \left(u_i', u_{i+1}', \dots, u_{i+k-1}'\right) \qquad [12a]$$

$$d(u_i\, u_{i+1}\, \dots\, u_{i+k-1}\;,\; u_i'\, u_{i+1}'\, \dots\, u_{i+k-1}') \leq \epsilon \,. \qquad [12b]$$

The number of strings of k gates satisfying the last condition is given by $\Omega_\epsilon(\mathbb{1}, 2k)$.

B - Extensivity of circuit entropy at the complexity threshold. Here we argue that the circuit entropy $\mathcal{S}(P,\mathcal{K}(P))$ for circuits of minimum length is extensive in the circuit complexity $\mathcal{K}(P)$. The upper bound $\mathcal{S}(P,\mathcal{K}(P)) \leq \mathcal{K}(P)\ \log_2 |G|$ derived from Eq. (5) of the main text establishes that the circuit entropy is at most extensive in $\mathcal{K}(P)$. Below we argue that $\mathcal{S}(P,\mathcal{K}(P))$ is also bounded below by a term proportional to $\mathcal{K}(P)$. For concreteness, we use the gate set G comprised of 3-bit permutation gates in S_8, with $|G| = 8!\binom{n}{3}$. We consider circuits for which all inputs are affected by at least one gate, i.e., none of the n bitlines are untouched by the circuit.

We proceed by considering the outputs of each of the $\mathcal{K}(P)$ gates in the circuit. Each of these outputs are either: (a) connected to the inputs of another gate; or (b) exit unimpeded as output bitlines of the circuit. In case (a) we have an internal link between the two gates. There are $3 \times \mathcal{K}(P)$ outputs originating from the $\mathcal{K}(P)$ 3-bit gates, which also include the exactly n outputs of gates that connect all the way to the end of the circuit. As a result, the number of internal links within the circuit is given by $N_\ell = 3 \times \mathcal{K}(P) - n$. For each of these internal links one can negate the output of the gate that acts before and the input of the gate that acts after, thus obtaining different gates that are part of a circuit that yields the same P. These negations can be implemented or not for each of the internal links, which leads to a degeneracy of at least 2^{N_ℓ}. For the polynomially-sized programs of interest in this paper, $\mathcal{K}(P) \geq n$, and we thus obtain

$$S(P,\mathcal{K}(P)) \geq 3\mathcal{K}(P) - n \geq 2\mathcal{K}(P) \,. \qquad [13]$$

Together with upper bound of Eq. (5) in the main text, the above lower bound establishes the extensively of $S(P,\mathcal{K}(P))$ with $\mathcal{K}(P)$, which we express generically as,

$$\mathcal{S}(P,\mathcal{K}(P)) \approx \gamma(P;G)\ \mathcal{K}(P) \,, \qquad [14]$$

where the dependence on the specific permutation P and the gate set G are made explicit in the coefficient $\gamma(P;G)$. In writing this expression we tacitly assume that the argument given above will go through for any universal gate set G.

We note that the entropy computed above is associated with an internal redundancy that arises through a “gauge" transformation that is absorbed in redefining two gates that share a link. Beyond this minimum set of redundancies, which occurs for any arrangement of gates, there are other “structural" sources of entropy associated with different gate arrangements or circuit architectures that yield functionally equivalent circuits.

C - Model for the dependence of the circuit entropy on the depth and complexity of computation. In this section we present a derivation of the circuit entropy $\sigma(\mathcal{K},\mathcal{N})$ by using a random walk model for the change in complexity with the addition of gates, which should provide further intuition about the discussion and results of the main text. This toy model is based on the observation that, upon adding a single gate, the circuit complexity increases or decreases by at most one. To see this, consider the permutation obtained by adding a single gate g to a permutation P. Using the inequality for complexities, namely $\mathcal{K}(P_1P_2) \leq \mathcal{K}(P_1) + \mathcal{K}(P_2)$, and the condition $\mathcal{K}(g) = \mathcal{K}(g^{-1}) = 1$, we immediately obtain, $\mathcal{K}(Pg) \leq \mathcal{K}(P) + \mathcal{K}(g) = \mathcal{K}(P) + 1$, and $\mathcal{K}(P) \leq \mathcal{K}(Pg) + \mathcal{K}(g^{-1}) = \mathcal{K}(Pg) + 1$. Thus complexity can only change by ± 1 or 0, as claimed, i.e.,

$$-1 \leq \mathcal{K}(Pg) - \mathcal{K}(P) \leq +1 \,. \qquad [15]$$

Next consider $P = g_1\, g_2\, \dots\, g_\mathcal{N}$, a circuit of $\mathcal{N}$ gates, and imagine the evolution of the partial complexity $k = \mathcal{K}(g_1\, g_2\, \dots\, g_n)$ after only the first $n \leq \mathcal{N}$ gates are applied. The evolution of k with n is a walk that starts at $(n,k) = (0,0)$ and ends at $(\mathcal{N},\mathcal{K})$, with the constraint that all intermediate steps satisfy $k \geq 0$.

For a random circuit, the evolution is a random walk that at each step – corresponding to the addition of one gate, or $\Delta n = 1$ – changes complexity by $\Delta k = \pm 1, 0$ with probabilities $p_\pm, p_0$. During the initial growth of the circuit with n, i.e, the early steps of the walk, we expect that these probabilities would depend on history; however, we assume that $p_\pm, p_0$ reach a steady state as the circuit length grows, with values that only depend on the gate set G. We further posit that the number of

(random) circuits of $\mathcal{N}$ gates and complexity $\mathcal{K}$ is proportional to the number of walks that start at $(n,k)=(0,0)$ and end at $(\mathcal{N},\mathcal{K})$, properly weighted by the probabilities $p_\pm$ and p_0. We proceed with the computation of the number of such walks.

Let $n_\pm$, n_-, and n_0 denote the number of steps involving complexity changes by $\Delta k = \pm 1$ and 0, respectively, which, in turn satisfy the constraints $\mathcal{N} = n_+ + n_- + n_0$ and $\mathcal{K} = n_+ - n_-$. The number of such walks is given by $\binom{\mathcal{N}}{n_0}\, C(\mathcal{N}-n_0,\mathcal{K})$, where the first factor counts the number of ways of placing steps with $\Delta k = 0$, and the second factor counts the number of walks of n_+ “up"-steps and n_- “down"-steps with $n_+ + n_- = \mathcal{N} - n_0$ that end at $\mathcal{K}$ while never crossing the $k=0$ line (since complexity cannot be negative). $C(n,k)$ can be determined via a variant of the problem of computing Catalan numbers (because of the restriction that $k \geq 0$ for all intermediate steps); it satisfies the recursion

$$C(n,k) = C(n-1,k-1) + C(n-1,k+1)\ , \tag{16}$$

where $C(n,0) = \binom{n}{n/2} - \binom{n}{n/2+1}$ is a Catalan number that counts the number of balanced non-negative walks, i.e., walks that start and end at $k=0$ after n (even) steps but that can only explore points with $k \geq 0$. By using Pascal’s triangle relations it is then easy to see that the solution to the recursion relation of Eq. (16) is given by

$$C(n,k) = \binom{n}{\frac{1}{2}(n+k)} - \binom{n}{\frac{1}{2}(n+k+2)}\ . \tag{17}$$

[For example, one can easily check that $C(k,k)=1$ consistent with the fact that there is only one walk that can reach complexity k after k steps, i.e., after a walk with $n_+ = k$ and $n_- = 0$.]

We can thus express the number of valid walks as

$$\binom{\mathcal{N}}{n_0} C(\mathcal{N}-n_0,\mathcal{K}) = \frac{\mathcal{N}!}{n_+!\, n_-!\, n_0!}\ \frac{n_+ - n_- + 1}{n_+ + 1}\ . \tag{18}$$

We are now in position to compute the number of (random) circuits of $\mathcal{N}$ gates of complexity $\mathcal{K}$ by summing over the appropriately weighted walks that start at $(n,k)=(0,0)$ and end at $(\mathcal{N},\mathcal{K})$:

$$\omega(\mathcal{K},\mathcal{N}) = \frac{1}{Z}\sum_{n_0} \frac{\mathcal{N}!}{n_+!\, n_-!\, n_0!}\ \frac{n_+ - n_- + 1}{n_+ + 1}\ p_+^{n_+}\, p_-^{n_-}\, p_0^{n_0}\ \delta_{\mathcal{N},n_+ + n_- + n_0}\ \delta_{\mathcal{K},n_+ - n_-}\ , \tag{19}$$

where Z is a normalization determined from the condition $\sum_{\mathcal{K}} \omega(\mathcal{K},\mathcal{N}) = |G|^{\mathcal{N}}$.

Let us consider the expression Eq. (19) with $p_+ \gg p_-, p_0$, a limit which is justified because for a generic circuit one expects that, upon addition a new (random) gate the complexity is much more likely to increase than to decrease or even remain unchanged. Also, in this limit $n_+ \gg n_-, n_0$ and $\frac{n_+ - n_- + 1}{n_+ + 1} \to 1$, leading to the following expression for the normalization,

$$\begin{aligned} Z &\approx \frac{1}{|G|^{\mathcal{N}}} \sum_{n_+,n_-,n_0} \frac{\mathcal{N}!}{n_+!\, n_-!\, n_0!}\ p_+^{n_+}\, p_-^{n_-}\, p_0^{n_0}\ \delta_{\mathcal{N},n_+ + n_- + n_0} \\ &\approx \frac{1}{|G|^{\mathcal{N}}}\ (\,p_+ + p_- + p_0)^{\mathcal{N}}\ . \end{aligned} \tag{20}$$

We have not replaced $p_+ + p_- + p_0$ by unity in order to compute the expectation value as well as the fluctuations in the complexity $\mathcal{K}$ from derivatives of $\ln Z$ with respect to the appropriate probabilities. More precisely,

$$\bar{n}_\pm = p_\pm \frac{\partial}{\partial p_\pm} \ln Z = \mathcal{N}\, p_\pm \quad \text{and} \quad \bar{n}_0 = p_0 \frac{\partial}{\partial p_0} \ln Z = \mathcal{N}\, p_0\ , \tag{21}$$

from which it follows that the average complexity is given by:

$$\overline{\mathcal{K}} = \bar{n}_+ - \bar{n}_- = (p_+ - p_-)\ \mathcal{N}\ , \tag{22}$$

and the corresponding fluctuations in the complexity by:

$$\overline{\mathcal{K}^2} - \overline{\mathcal{K}}^2 = [p_+(1-p_+) + p_-(1-p_-) + 2p_+p_-]\ \mathcal{N}\ . \tag{23}$$

These results recover the linear growth of the average complexity and the extensivity of the “specific heat" $C_{\mathcal{N}} = -(\partial\mathcal{K}/\partial T)|_{\mathcal{K}^*,\mathcal{N}}$ controlling complexity fluctuations.

This toy model also allows us to recover the lower bound on the entropy of the identity permutation $\mathbb{1}$, which corresponds to $\mathcal{K}=0$ and for which $\omega(0,\mathcal{N}) = \Omega(P=\mathbb{1},\mathcal{N})$ and $\bar{S}(\mathcal{K}(P)=0,\mathcal{N}) = \sigma(\mathcal{K}=0,\mathcal{N})$. The latter equality follows since there is only one functionality with $\mathcal{K}=0$, namely the identity. Zero complexity corresponds to $n_+ = n_- = \frac{1}{2}(\mathcal{N}-n_0)$, in which case the expression for $\omega(0,\mathcal{N})$ reduces to

$$\omega(0,\mathcal{N}) = \frac{1}{Z}\sum_{n_0} \binom{\mathcal{N}}{n_0} C(\mathcal{N}-n_0,0)\ p_+^{(\mathcal{N}-n_0)/2}\, p_-^{(\mathcal{N}-n_0)/2}\, p_0^{n_0}\ . \tag{24}$$

Using the asymptotic limit of the Catalan number $C(\mathcal{N}-n_0,0) \approx \frac{2^{\mathcal{N}-n_0}}{\sqrt{\pi/8}\,(\mathcal{N}-n_0)^{3/2}}$ allows us to perform the (binomial) sum over n_0, which yields

$$\begin{aligned}\omega(0,\mathcal{N}) &= \frac{1}{Z}\,\eta(\mathcal{N})\,\left(2\sqrt{p_+\,p_-}+p_0\right)^{\mathcal{N}}\\ &= \frac{1}{Z}\,\eta(\mathcal{N})\,\left[1-(\sqrt{p_+}-\sqrt{p_-})^2\right]^{\mathcal{N}}\ ,\end{aligned} \tag{25}$$

where the factor $\eta(\mathcal{N}) \approx 1/(\sqrt{\pi/8}\,\mathcal{N}^{3/2})$ is only a polynomial correction. Thus, up to a subextensive term in $\mathcal{N}$,

$$\sigma(0,\mathcal{N}) = \bar{S}(0,\mathcal{N}) = \mathcal{N}\ \log|G| + \mathcal{N}\ \log[1-(\sqrt{p_+}-\sqrt{p_-})^2] + \dots\ . \tag{26}$$

The second term in Eq. (26), which depends on the probabilities, is negative and thus one immediately obtains the upper bound $\bar{S}(0,\mathcal{N}) \le \mathcal{N}\ \log|G|$. The lower bound for $\bar{S}(0,\mathcal{N})$ is derived by noticing that the probability that the complexity decreases satisfies the bound $p_- \ge 1/|G|$, to account for the fact that there is at least one gate in the set G that, when added, lowers the complexity, namely the inverse of the last gate of the circuit (prior to the addition). That minimum value of p_- implies that $p_+ \le 1-1/|G|$. We can thus bound the second term in Eq. (26):

$$\begin{aligned}\mathcal{N}\ \log[1-(\sqrt{p_+}-\sqrt{p_-})^2] &\ge \mathcal{N}\ \log\left[1-\left(\sqrt{(1-1/|G|)}-\sqrt{1/|G|}\right)^2\right]\\ &\ge \mathcal{N}\ \log[2\sqrt{(1-1/|G|)}\,\sqrt{1/|G|})]\\ &\ge -\frac{1}{2}\,\mathcal{N}\ \log|G|\ .\end{aligned} \tag{27}$$

Eq. (26) then reduces to: $\bar{S}(0,\mathcal{N}) \ge \mathcal{N}\ \log|G| - \frac{1}{2}\,\mathcal{N}\ \log|G| = \frac{1}{2}\,\mathcal{N}\ \log|G|$, which recovers the lower bound for the entropy of circuits implementing the identity permutation that we derived in the body of the paper by other means.
